# New York State Legislation

**Published:** 1897-02

**Authors:** 


### EDITORIAL.

### NEW YORK STATE.

ANOTHER effort to amend the law in New York State extending the term of registration to January 1, 1898, has loomed up in the Assembly at Albany. It also adds a new feature, *any branch thereof*, looking to the favor of perhaps so-called veterinary dentists and dehorners, both of whom have suffered the penalty of practising without a proper license. Eternal vigilance is to be the price of maintaining this wise Act, and it calls forth again from every true veterinarian the most emphatic protest. Let these men, who are certainly not in these days sufficiently well equipped as to be called for by any great public need, but who wish to tear down wise and proper safeguards for the people of New York State, enter some one of the several excellent schools in the State and thus enter the profession legitimately and properly prepared to perform the duties thereof. SCOTCH THIS SNAKE LEGISLATION.

Assembly bill No. 231, creating a State Veterinarian, makes said appointee a member of the State Board of Health, thus removing this office from control of the Department of Agriculture. On the whole, the change is judicious, inasmuch as the prevalence of contagious and infectious diseases among animals bears an important relation to public health; there are likely also to be a closer unity of interests and avoidance of the friction prejudicial to the good interests to be served in public sanitary measures. Empire State veterinarians will have much to engross their attention during the present session of their Legislature. One factor of great importance must not be forgotten, and that is the danger of estranging the agriculturists from the veterinary profession, from whom the latter receive so large a measure of support.

Assembly Bill No. 25 should never be allowed to emerge from the Judiciary Committee. It is special legislation of the worst kind, and will establish a bad precedent. Whatever rights or privileges one Charles McCormick may have under the original Act he neglected or deliberately forfeited by failing to take advantage in proper season of the privileges granted him, and therefore any injury he may suffer is of his own production. One bill or enabling Act of this kind will open the floodgates for a perfect avalanche of such concessions. Every veterinarian in the Empire State who prizes the safeguards obtained should act promptly and earnestly in defeating this measure.

